# Supplementary material for: Gingival solitary chemosensory cells are immune sentinels for periodontitis
Source: Nat Commun. 2019 Oct 3;10:4496. doi: 10.1038/s41467-019-12505-x (PMC6776549; doi:10.1038/s41467-019-12505-x)
Supplement: Supplementary file 1 — Supplementary Figures and Tables [file 41467_2019_12505_MOESM1_ESM.pdf]

SUPPLEMENTAL INFORMATION

Gingival Solitary Chemosensory Cells Are Immune Sentinels for Periodontitis

Zheng et al.

Contents:

Supplementary Figures 1-6

Supplementary Tables 1-3

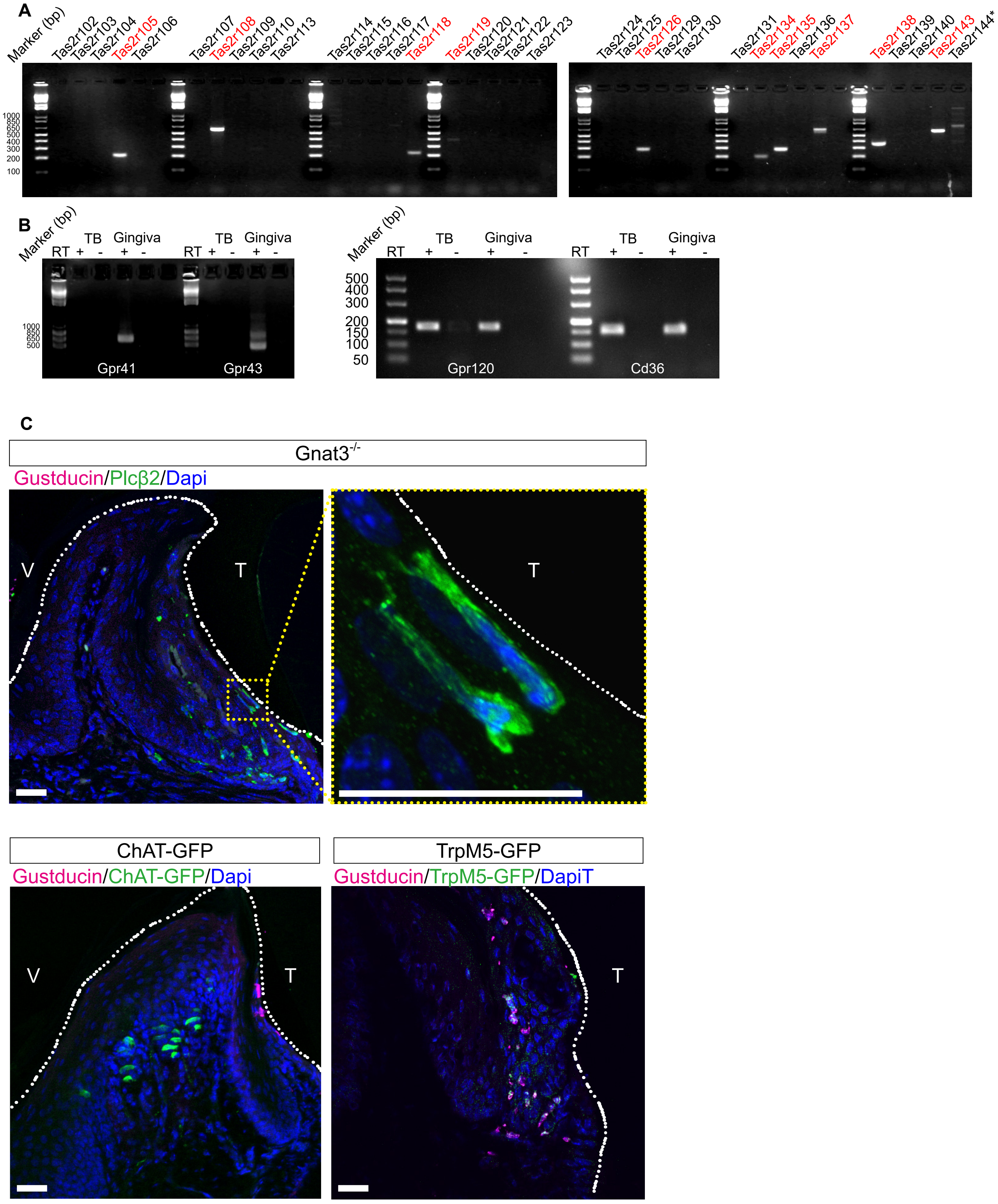

Supplementary Figure 1. Taste Signaling Molecules Expressed in Gingival Tissue (Related to Figure 1)
(A) RT-PCR was used to examine expression in mouse tissues of all 35 Tas2rs. The cDNA templates were derived from mouse gingival tissue. Tas2rs shown in red represent those expressed in gingiva. *The Tas2r144 band represents a non-specific PCR product with unexpected size.
(B) RT-PCR for Gpr41, Gpr43, Gpr120 and Cd36 in taste buds and gingiva. TB: taste buds; RT (+/-): with/without reverse transcription.
(C) Immunofluorescence staining for α-gustducin and Plcβ2 in gingival tissues from Gnat3$^{-/-}$, choline acetyltransferase (ChAT)-GFP, and TrpM5-GFP mice. The white dotted lines outline the tooth margins. T: the tooth facing side; V: the vestibular groove facing side. Yellow dotted lines indicate the fields with magnified views. Scale bars: 150 µm.
Source data are provided as a Source Data file.

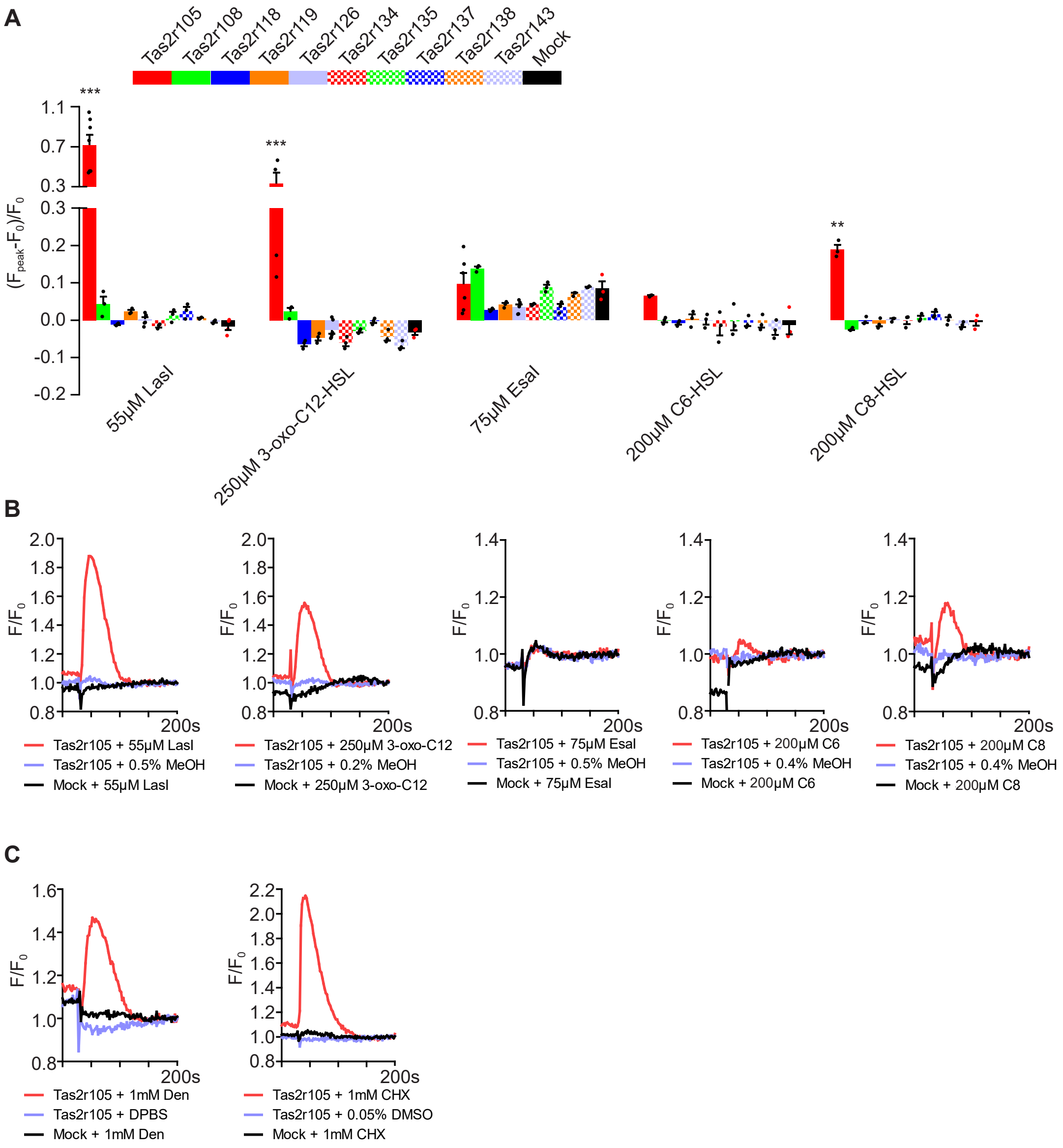

Supplementary Figure 2. Tas2r105 Responded to Acylated Homoserine Lactones (AHLs) (Related to Figure 1)
(A) HEK293 cells were transfected with Gα16Gust44 plus one of the 10 Tas2rs found to be expressed in gingival tissue, or with empty vector (Mock). Cells were loaded with Fluo-4 calcium dye and then stimulated with five bacterially produced or synthetic AHLs. Fluorescence intensity changes indicating intracellular calcium concentrations were monitored with a FlexStation 3. Data are presented as means ± SEM (n=3 independent experiments, except some assays where n=4, 6 or 7 independent experiments, see details in Source Data file). **, $p < 0.01$, ***, $p < 0.001$; one-way ANOVA test followed by Dunnett's test to compare experimental groups with mock control.
(B) Representative calcium response curves of HEK293 cells expressing Tas2R105 and Gα16Gust44 to AHLs. MeOH, a control group with methanol at the corresponding concentration as in the AHL stimuli. Representative data of 3 independent experiments.
(C) Calcium response curves of HEK293 cells expressing Tas2r105 and Gα16Gust44 to denatonium benzoate (Den) and cycloheximide (CHX). Vehicle control groups comprised Dulbecco's PBS (DPBS) or 0.05% DMSO at the corresponding concentration as in the stimuli. Representative data of 3 independent experiments.
Source data are provided as a Source Data file.

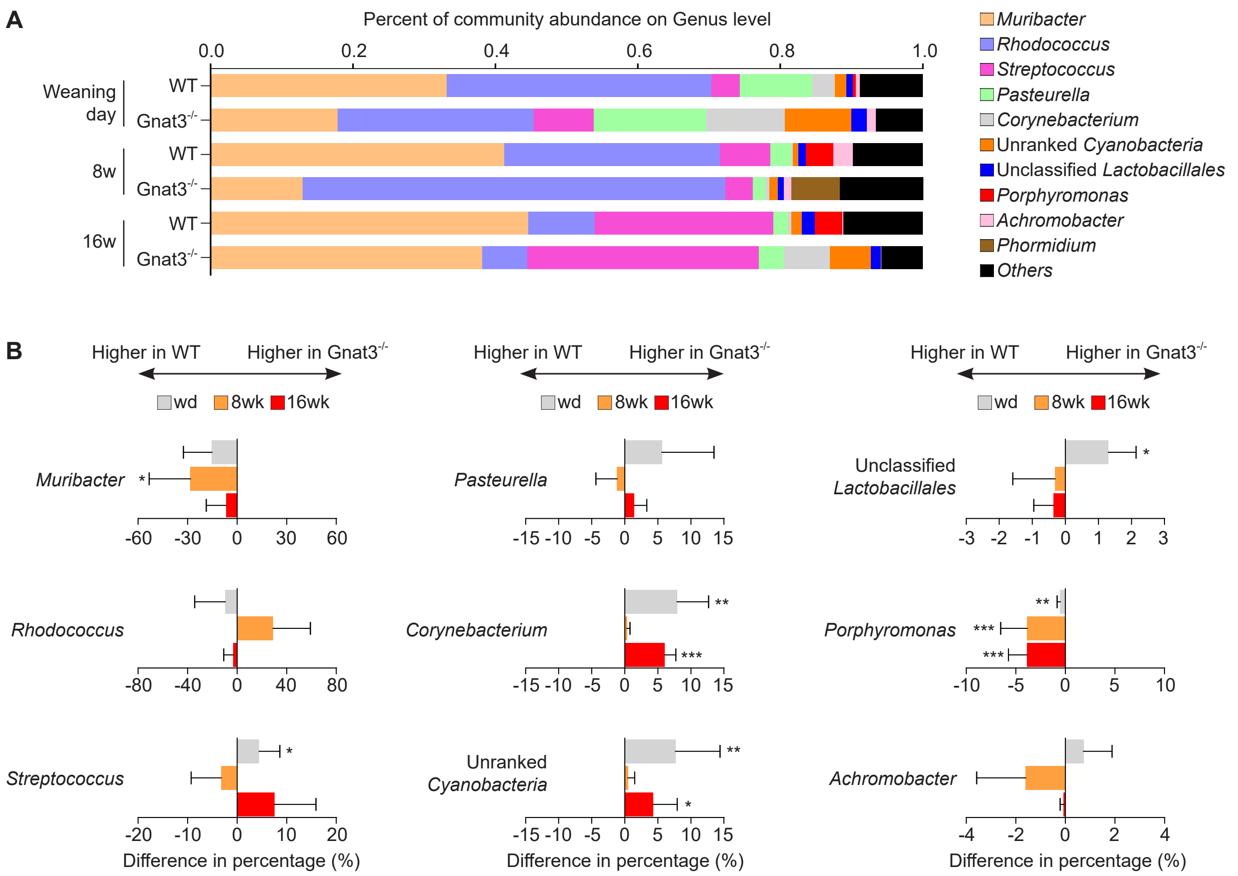

Supplementary Figure 3. The Commensal Oral Microbial Composition Differed between Wild-Type (WT) and Gnat3$^{-/-}$ Mice (Related to Figure 2)
(A) Percentage of community abundance at the genus level in WT and Gnat3$^{-/-}$ mice at weaning day (wd) and at 8 and 16 weeks (wk) of age.
(B) Percentage change of nine most abundant genera in WT and Gnat3$^{-/-}$ mice. Data are means ± 95% confidence interval (n=8 mice). *, $p < 0.05$, **, $p < 0.01$, ***, $p < 0.001$; Wilcoxon rank-sum test.
Source data are provided as a Source Data file.

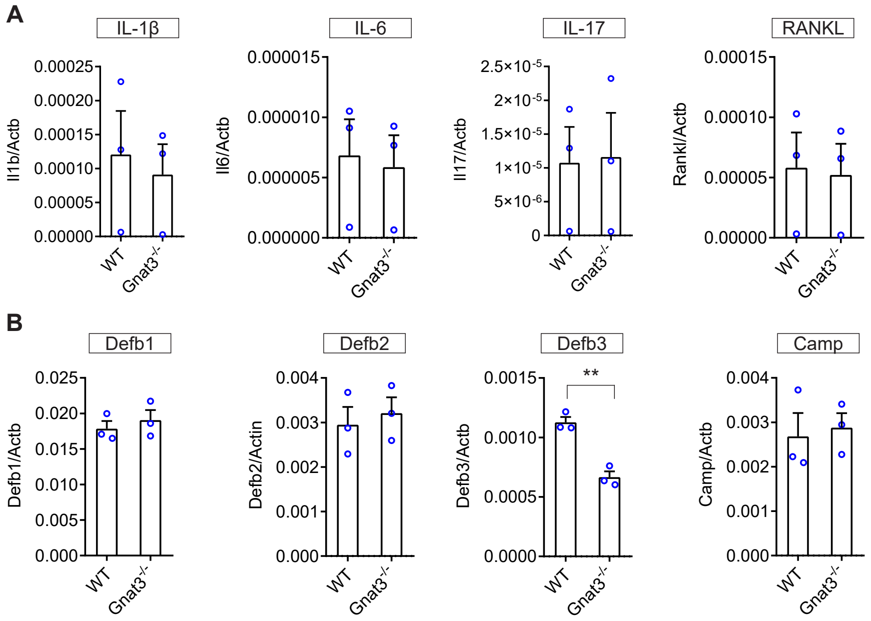

Supplementary Figure 4. Cytokine and Antimicrobial Peptide mRNA Expression from Gingival Tissue from the Unligatured Sides of WT and Gnat3$^{-/-}$ Mice (Related to Figure 3)

Expression of pro-inflammatory cytokines (A) and antimicrobial peptides (B) determined by qPCR. Results are normalized against β-actin mRNA expression (means ± SEM, n=3 independent experiments). IL-1β, -6, -17: interleukin-1β, -6, -17, respectively; RANKL: receptor activator of nuclear factor kappa-B ligand; Defb1-3: β-defensin 1-3, respectively; Camp: cathelicidin antimicrobial peptide LL-37. **, $p < 0.01$, Student's t test.

Source data are provided as a Source Data file.

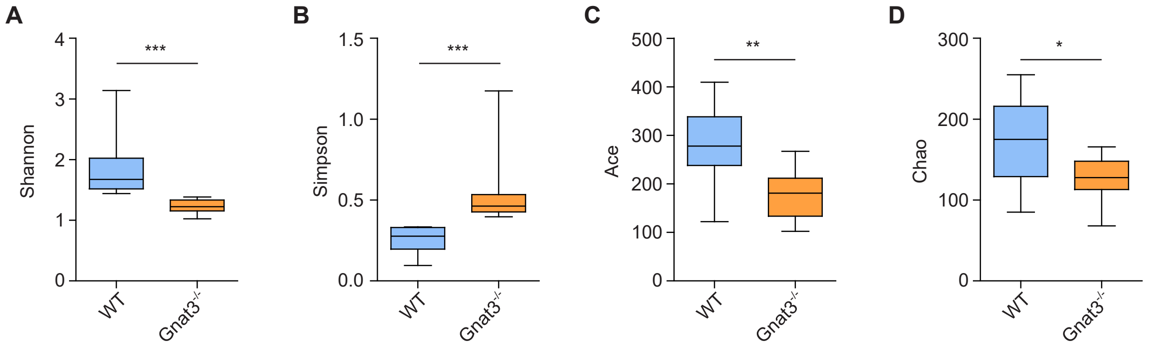

Supplementary Figure 5. The Alpha Diversity of Ligature Microbiota Was Lower in Gnat3$^{-/-}$ Mice (Related to Figure 4)
The α diversity of the ligature microbiome was compared between wild-type (WT) and Gnat3$^{-/-}$ mice using Shannon (A), Simpson (B), Chao (C), and Ace (D) indices. The Shannon index is positively correlated with α diversity, while the Simpson index is negatively correlated. Ace and Chao indices, representing the operational taxonomic units in samples, are also positively correlated with α diversity. Data are presented as standard box plots, with the boxes presenting the first and third quartiles and the whiskers representing the 5th and 95th percentiles (n=10 mice). *, $p < 0.05$, **, $p < 0.01$, ***, $p < 0.001$; Wilcoxon rank-sum test.
Source data are provided as a Source Data file.

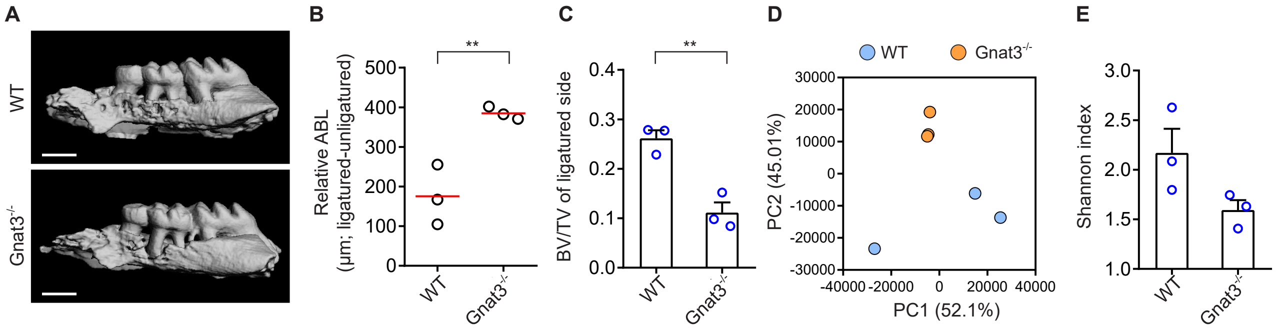

Supplementary Figure 6. Ligature-Induced Periodontitis Is More Severe in Cohoused Mice Lacking Gnat3 (Related to Figure 3 and 4)
(A) Ligatured maxillae from cohoused WT and Gnat3$^{-/-}$ mice. Scale bars: 500 μm.
(B) Quantitation of relative alveolar bone loss (ABL) calculated by subtracting the ABL of the unligatured side from the ABL of the ligatured side. Results for each mouse are plotted; the red lines indicate the means (n=3 mice). **, $p < 0.01$, Student's t test.
(C) MicroCT analysis of alveolar bone (n=3 mice). BV/TV: bone volume/tissue volume. **, $p < 0.01$, Student's t test.
(D) Principal component analysis (PCA) of microbiota recovered from ligatures around molars of WT and Gnat3$^{-/-}$ mice (n=3 mice). Each circle represents an individual ligature sample, colored by genotype.
(E) The Shannon index (positively correlated with α diversity) of the ligature microbiome was compared between WT and Gnat3$^{-/-}$ mice.
Error bars in C and E represent the SEM.
Source data are provided as a Source Data file.

Supplementary Table 1. Key Resources (Related to Figures 1-5).

REAGENT or RESOURCE	SOURCE	IDENTIFIER
Antibodies		
Rabbit anti-α-gustducin antibody	Santa Cruz	Cat. no. SC-395, RRID: AB_673678
Goat anti-Plcβ2 antibody	Santa Cruz	Cat. no. SC-31759, RRID: AB_2163242
Chicken anti-GFP antibody	Millipore	Cat. no. AB16901, RRID: AB_11212200
Alex Fluor 594 donkey anti-rabbit IgG	Molecular Probes	Cat. no. A-21207, RRID: AB_141637
Alex Fluor 488 donkey anti-goat IgG	Molecular Probes	Cat. no. A-11055, RRID: AB_142672
Alex Fluor 488 donkey anti-chicken IgY	Jackson ImmunoResearch	Cat. no. 703-546-155, RRID: AB_2340376
Bacterial Strains		
One Shot™ TOP10 Chemically Competent *E. coli*	Thermo Fisher Scientific	Cat. no. C404003
Biological Samples		
Murine gingival tissues	This study	N/A
Murine taste buds	This study	N/A
Murine maxillae	This study	N/A
Murine oral swabs (microbiota)	This study	N/A
Recovered ligatures (microbiota)	This study	N/A
Chemicals, Peptides, and Recombinant Proteins		
LasI	Churchill Lab., Univ. Colo. Denver	
EsaI	Churchill Lab., Univ. Colo. Denver	
3-oxo-C12-HSL	Sigma-Aldrich	Cat. no. 09139
C6-HSL	Sigma-Aldrich	Cat. no. 09926
C8-HSL	Sigma-Aldrich	Cat. no. 10940
Denatonium benzoate	Sigma-Aldrich	Cat. no. 52487
Cycloheximide	Sigma-Aldrich	Cat. no. C7698
Isoproterenol	Sigma-Aldrich	Cat. no. 1351005
Critical Commercial Assays		
Dispase II	Roche	Cat. no. 04942078001
Collagenase A	Roche	Cat. no. 10103578001
PureLink™ RNA Mini Kit	Thermo Fisher Scientific	Cat. no. 12183025
RQ1 RNase-Free DNase	Promega	Cat. no. M6101
SuperScript™ VILO™ Master Mix	Thermo Fisher Scientific	Cat. no. 11755050
Platinum™ *Taq* DNA Polymerase	Thermo Fisher Scientific	Cat. no. 10966026
pcDNA™3.1/Zeo$^{(+)}$	Thermo Fisher Scientific	Cat. no. V86020
Lipofectamine 2000	Thermo Fisher Scientific	Cat. no. 11668019
Fluo-4	Thermo Fisher Scientific	Cat. no. F14201
Fura-2	Thermo Fisher Scientific	Cat. no. F14185
PurFlock™ Ultra Sterile Flocked Swabs	Puritan	Cat. no. 253318U BT

PureLink™ Microbiome DNA Purification Kit	Thermo Fisher Scientific	Cat. no. A29790
Pico-Green kit	Thermo Fisher Scientific	Cat. no. P11496
6-0 silk ligatures	Fine Science Tools	Cat. no. 1802060
TaqMan Fast Universal Master Mix	Applied Biosystems	Cat. no. 4444557
Fast SYBR Green Master Mix	Applied Biosystems	Cat. no. 4385612
Deposited Data		
16S rDNA sequencing raw data	http://www.ncbi.nlm.nih.gov/Traces/sra	Acc. no. SRP126006.
Experimental Models: Cell Lines		
Human embryonic kidney 293 (HEK293) PEAKrapid cells	ATCC	Cat. no. CRL-2828
Experimental Models: Organisms/Strains		
Wild-type mice (C57BL/6J background)	Monell Chemical Senses Center	
Gnat3$^{-/-}$ mice (C57BL/6J background)	Monell Chemical Senses Center	
Pou2f3$^{-/-}$ mice (C57BL/6J background)	From Dr. Ichiro Matsumoto (Monell Chemical Senses Center)	
ChAT-GFP mice (FVB/N background)	From Sukumar Vijayaraghavan (Univ. Colo. Denver)	
TrpM5-GFP mice (C57BL/6J background)	Monell Chemical Senses Center	
Recombinant DNA		
pcDNA™3.1/Hygro$^{(+)}$ -Gα16gust44	From P.J.	
pcDNA™3.1/Zeo$^{(+)}$ -RSST-Tas2r105	This study	
pcDNA™3.1/Zeo$^{(+)}$ -RSST-Tas2r108	This study	
pcDNA™3.1/Zeo$^{(+)}$ -RSST-Tas2r118	This study	
pcDNA™3.1/Zeo$^{(+)}$ -RSST-Tas2r119	This study	
pcDNA™3.1/Zeo$^{(+)}$ -RSST-Tas2r126	This study	
pcDNA™3.1/Zeo$^{(+)}$ -RSST-Tas2r134	This study	
pcDNA™3.1/Zeo$^{(+)}$ -RSST-Tas2r135	This study	
pcDNA™3.1/Zeo$^{(+)}$ -RSST-Tas2r137	This study	
pcDNA™3.1/Zeo$^{(+)}$ -RSST-Tas2r138	This study	
pcDNA™3.1/Zeo$^{(+)}$ -RSST-Tas2r143	This study	
Software and Algorithms		
Image J	NIH	https://imagej.nih.gov/ij/
Image pro plus 6.0	Media Cybernetics	
Usearch version 7.1	Drive5	http://drive5.com/uparse/
I-Sanger	Majorbio co.	http://www.i-sanger.com/
Prism software	GraphPad Software Inc.	

Supplementary Table 2. Analysis of Microbiome Data (Related to Figures 2 and 4).

Sample type	Comparison groups	ANOSIM (p value)	Adonis (p value)
Oral swab	WT: wd vs. 8wk vs. 16wk (see Fig. 2 F)	0.006	0.02
	$Gnat3^{-/-}$: wd vs. 8wk vs. 16wk (see Fig. 2 G)	0.001	0.001
	WT, wd vs. $Gnat3^{-/-}$, wd (see Fig. 2 H)	0.001	0.001
	WT, 8wk vs. $Gnat3^{-/-}$, 8wk (see Fig. 2 I)	0.038	0.042
	WT, 16wk vs. $Gnat3^{-/-}$, 16wk (see Fig. 2 J)	0.002	0.001
Ligature	WT vs. $Gnat3^{-/-}$ (see Fig. 3F)	0.001	0.001

Abbreviations: ANOSIM = analysis of similarities; Adonis = multivariate analysis of variance; wd = weaning day.

Supplementary Table 3. Sequences of Primers and Probes (Related to Figures 1, 3-5).

Name	Sequence (5'-3')	Name	Sequence (5'-3')	Name	Sequence (5'-3')
**Gnat3_131F**	GAGAGCAAGGAATCAGCCAG	**Tas2r119_276F**	TCTGGTTTGCCACATGGCTT	**Tas2r143_99F**	AGAGTGGATGAGGAACCGGA
**Gnat3_252R**	GTGCTTTTCCCAGATTCACC	**Tas2r119_658R**	GGCATGTCTGCTAGGTTCCC	**Tas2r143_683R**	GCCATGGTATGTGCCTGAGT
**Trpm5_952F**	TTCCCCAGCGAGTGTTTCTC	**Tas2r120_282F**	CACTTGGCTGGGGACCATAC	**Tas2r144_651F**	CTCACTCAAGAGGCACACCC
**Trpm5_1221R**	CCATTCCACGTCCCCATTGA	**Tas2r120_669R**	GTGGACCATGGTGCTCTGAT	**Tas2r144_757R**	TGAGAGAGTGGCTGGTCGAT
**Plcb2_1954F**	CCTGGAGGTGACAGCTTATGA	**Tas2r121_658F**	CGAGACCCCAGCACTAAAGC	**Gpr41_F**	CAGAGTGCCAGTTGTCCAATA
**Plcb2_2078R**	GCTCCGTGAAGGAAGAGACA	**Tas2r121_888R**	CATCACCCAAAGACTGGCTTG	**Gpr41_R**	ATGCCAGGAACCAACAGACT
**Tas2r102_26F**	AGGCGACGCTGTTATATGCC	**Tas2r122_181F**	CAACAATTGCTGGTGCCTCT	**Gpr43_F**	CAAACTCGGGATGCTTCAG
**Tas2r102_354R**	AAGCCAGAGGCTGAAGTGAC	**Tas2r122_771R**	GGAGCTTGCCACAATAAGCA	**Gpr43_R**	AGCAGCAACAGGAGCAAGTC
**Tas2r103_565F**	ACCCCATTCGCTGTGTCTTT	**Tas2r123_102F**	AGTGAACATCATGGACTGGGT	**Gpr120_F**	GGGGACCAGGAAATTCCGATT
**Tas2r103_877R**	AGGCTTGCCTCAGCTTACTG	**Tas2r123_249R**	TCTCCTAGGCAAATGTGGGC	**Gpr120_R**	CGCGATGCTTTCGTGATCTG
**Tas2r104_434F**	TTCCGCTAGCTGTGAAGGTC	**Tas2r124_393F**	GCTTCTGGGAAGCTTGGTGT	**Cd36_F**	GCAGCCTCCTTTCCACCTTT
**Tas2r104_881R**	AGTGCCCTCATAGTGGCTTG	**Tas2r124_681R**	ATTTCTGTGGGCCGTAGCAC	**Cd36_R**	TGTCTGGATTCTGGAGGGGT
**Tas2r105_294F**	GTTTGCCACCAGCCTAAGCA	**Tas2r125_666F**	CACCACCACAGCTGCACATA	**Actb_251F**	GGTCAGAAGGACTCCTATGTGG
**Tas2r105_506R**	TCCCAGTACATCTCCGAGGTC	**Tas2r125_935R**	CAGGGAACCAACATCCGTACA	**Actb_353R**	TGTCGTCCCAGTTGGTAACA
**Tas2r106_2F**	TGCTGACTGTAGCAGAAGGA	**Tas2r126_140F**	TCCTCTTCAGTTTGGGCACC	**Defb3_56F**	TGTCTCCACCTGCAGCTTTTAG
**Tas2r106_134R**	AAGCCAGCTGTGGAGAACTT	**Tas2r126_424R**	CGGACACCAAGATAGAGCCC	**Defb3_166R**	AACTGCCAATCTGACGAGTGT
**Tas2r107_119F**	GCTCGGAGTTTTAGGGGACA	**Tas2r129_671F**	TTGCAGATGCCCACATCAGA	**Defb2_86F**	CCACTCCAGCTGTTGGAAGTTT
**Tas2r107_873R**	AGAGGCATGTGGCTGTCAAA	**Tas2r129_818R**	GCTGCAACAATCTCGCAGAA	**Defb2_221R**	TTCTCTGGGAAACAGCTCCC
**Tas2r108_112F**	AGTCGCAGAATTGCCTCTCC	**Tas2r130_37F**	GCTGTTGGTGAGGCCTTAGT	**Defb1_151F**	GGAGCCAGGTGTTGGCATTC
**Tas2r108_688R**	GCCTCATAGCACCCATGTGA	**Tas2r130_546R**	GACAGAGGCATGTCCAGCTT	**Defb1_305R**	AGCTCTTACAACAGTTGGGCT
**Tas2r109_587F**	CTGTCCCCGTTGTTTTGTCC	**Tas2r131_314F**	CCCACATTTCCCATCCCCTT	**Camp_381F**	TCAGCTGTAACGAGCCTGGTG
**Tas2r109_915R**	CAACACAGAGAGAGAGGCGT	**Tas2r131_618R**	GTCAAGGCTTCGGGAGTGTT	**Camp_556R**	GCCAAGGCAGGCCTACTAC
**Tas2r110_700F**	CAGGTCAATGCCAAACCACC	**Tas2r134_457F**	ATGGCGGCCTGTGAAAACTA	**Il1b_119F**	TGCCACCTTTTGACAGTGATG
**Tas2r110_969R**	GCACCTCAGACAATGCAACA	**Tas2r134_663R**	GTGAGCCTGGGTGCTGTAAT	**Il1b_338R**	AAGGTCCACGGGAAAGACAC
**Tas2r113_632F**	ATATGCAGCACACCGCCAAA	**Tas2r135_543F**	GAGTGGCCATCAACCTTGGA	**Il6_187F**	GAGGATACCACTCCCAACAGACC
**Tas2r113_811R**	CCAGAGCCCAGACAAACAAA	**Tas2r135_830R**	GCAGAACTGAGTACCAGCGT	**Il6_327R**	AAGTGCATCATCGTTGTTCATACA
**Tas2r114_7F**	AGCACAATGGAAGGTGTCCT	**Tas2r136_712F**	CCCAGTGCTTCAACCCACAT	**Il17_284F**	CCTGGACTCTCCACCGCAA
**Tas2r114_622R**	GCCTGCGATGTCTCCAAAGT	**Tas2r136_963R**	CCAGAACCTTGCTCTCACCT	**Il17_488R**	CCCACCAGCATCTTCTCGAC
**Tas2r115_692F**	AGACTGTGGTTGCCTTCCTC	**Tas2r137_19F**	ACAAGCAAGGATCAGGGTGG	**Rankl_368F**	CGAGCGCAGATGGATCCTAA
**Tas2r115_922R**	AGGTTTTCTCACGCTTGCAC	**Tas2r137_657R**	CAGAAGGTAGGCAACCAGGG	**Rankl_614R**	CCACATCCAACCATGAGCCT
**Tas2r116_569F**	TTGCTGTGTCACTGGTCACT	**Tas2r138_618F**	AGCTTTCCTGGTTTCCTCGG	**Uni_152F**	CGCTAGTAATCGTGGATCAGAATG
**Tas2r116_684R**	TCTGATGTGGGCCTTAGTGC	**Tas2r138_983R**	GGAGGAACCTTGTGGACTGG	**Uni_220R**	TGTGACGGGCGGTGTGTA
**Tas2r117_92F**	ATGGGTTCATGGTCCTGGTC	**Tas2r139_3F**	GGCTCAACCCAGCAACTACT	**Uni_177P**	CACGGTGAATACGTTCCCGGGC
**Tas2r117_560R**	AACACCTGCCTGTGACACTT	**Tas2r139_432R**	CCACAGAAGCCAGGGCATTA	**Nl1060_F**	ACGGGAGTAAAAGGATTTGAGGCTAAT
**Tas2r118_127F**	TCACCGGTGGAGACGATTCT	**Tas2r140_665F**	CCAGCACCACAGCCCATATT	**Nl1060_R**	AATATTCATACTCATATCGCCTCCTTA
**Tas2r118_356R**	CTCAGCCAGAGGAAGATGGG	**Tas2r140_847R**	TTAGGACACAAGAGTGGCCC		
