# Supplementary material for: Gingival solitary chemosensory cells are immune sentinels for periodontitis
Source: Nat Commun. 2019 Oct 3;10:4496. doi: 10.1038/s41467-019-12505-x (PMC6776549; doi:10.1038/s41467-019-12505-x)
Supplement: Supplementary file 3 — Reporting Summary [file 41467_2019_12505_MOESM3_ESM.pdf]

nature research

Corresponding author(s): Robert F. Margolskee
Last updated by author(s): Sep 10, 2019

Reporting Summary

Nature Research wishes to improve the reproducibility of the work that we publish. This form provides structure for consistency and transparency in reporting. For further information on Nature Research policies, see Authors & Referees and the Editorial Policy Checklist.

Statistics

For all statistical analyses, confirm that the following items are present in the figure legend, table legend, main text, or Methods section.

n/a	Confirmed	
☐	☒	The exact sample size (*n*) for each experimental group/condition, given as a discrete number and unit of measurement
☐	☒	A statement on whether measurements were taken from distinct samples or whether the same sample was measured repeatedly
☐	☒	The statistical test(s) used AND whether they are one- or two-sided<br>*Only common tests should be described solely by name; describe more complex techniques in the Methods section.*
☒	☐	A description of all covariates tested
☒	☐	A description of any assumptions or corrections, such as tests of normality and adjustment for multiple comparisons
☐	☒	A full description of the statistical parameters including central tendency (e.g. means) or other basic estimates (e.g. regression coefficient) AND variation (e.g. standard deviation) or associated estimates of uncertainty (e.g. confidence intervals)
☒	☐	For null hypothesis testing, the test statistic (e.g. *F*, *t*, *r*) with confidence intervals, effect sizes, degrees of freedom and *P* value noted<br>*Give P values as exact values whenever suitable.*
☒	☐	For Bayesian analysis, information on the choice of priors and Markov chain Monte Carlo settings
☒	☐	For hierarchical and complex designs, identification of the appropriate level for tests and full reporting of outcomes
☒	☐	Estimates of effect sizes (e.g. Cohen's *d*, Pearson's *r*), indicating how they were calculated

Our web collection on statistics for biologists contains articles on many of the points above.

Software and code

Policy information about availability of computer code

Data collection	no software was used
Data analysis	For 16S rDNA sequencing data, statistical analyses were performed with I-Sangers online tools (http://www.i-sanger.com/). The differences in beta diversity (revealed by PCA) within groups were compared with ANOSIM and Adonis; the alpha diversity data and genus-level microbial composition data were analyzed by Wilcoxon rank-sum test. Statistical analyses of other data were performed with Prism software.<br>Image J from NIH (https://imagej.nih.gov/ij/)<br>Image pro plus 6.0 from Media Cybernetics<br>Trimmomatic (Bolger et al., 2014)<br>FLASH (Magoc and Salzberg, 2011)<br>Usearch version 7.1 from Drive5 (http://drive5.com/uparse/)<br>I-Sanger from Majorbio co (http://www.i-sanger.com/)<br>Prism software from GraphPad Software Inc.

For manuscripts utilizing custom algorithms or software that are central to the research but not yet described in published literature, software must be made available to editors/reviewers. We strongly encourage code deposition in a community repository (e.g. GitHub). See the Nature Research guidelines for submitting code & software for further information.

Data

Policy information about availability of data

All manuscripts must include a data availability statement. This statement should provide the following information, where applicable:

- Accession codes, unique identifiers, or web links for publicly available datasets
- A list of figures that have associated raw data
- A description of any restrictions on data availability

Microbiome sequencing data have been deposited in public database Sequence Read Archive (http://www.ncbi.nlm.nih.gov/Traces/sra) with accession numbers SRP126006 and SRP215896. All other data generated or analyzed during this study are included in this published article (and its extended data files).

Field-specific reporting

Please select the one below that is the best fit for your research. If you are not sure, read the appropriate sections before making your selection.

☒ Life sciences ☐ Behavioural & social sciences ☐ Ecological, evolutionary & environmental sciences

For a reference copy of the document with all sections, see nature.com/documents/nr-reporting-summary-flat.pdf

Life sciences study design

All studies must disclose on these points even when the disclosure is negative.

Sample size	Sample sizes were chosen based on statistically significant results obtained in other publications that used similar methods.
Data exclusions	No data were excluded.
Replication	All experiments were independently replicated at least 3 times.
Randomization	In the topical treatment experiment, mice were randomly assigned to (a) the denatonium treatment group, or (b) the PBS control group.
Blinding	The investigators were blinded as to the strain of mice used (WT vs KO).

Reporting for specific materials, systems and methods

We require information from authors about some types of materials, experimental systems and methods used in many studies. Here, indicate whether each material, system or method listed is relevant to your study. If you are not sure if a list item applies to your research, read the appropriate section before selecting a response.

Materials & experimental systems

n/a	Involved in the study	
☐	☒	Antibodies
☐	☒	Eukaryotic cell lines
☒	☐	Palaeontology
☐	☒	Animals and other organisms
☒	☐	Human research participants
☒	☐	Clinical data

Methods

n/a	Involved in the study	
☒	☐	ChIP-seq
☒	☐	Flow cytometry
☒	☐	MRI-based neuroimaging

Antibodies

Antibodies used	Rabbit anti-α-gustducin antibody, Santa Cruz Biotechnology, Cat. no. SC-395, RRID: AB_673678<br>Goat anti-Plcβ2 antibody, Santa Cruz Biotechnology, Cat. no. SC-31759, RRID: AB_2163242<br>Chicken anti-GFP antibody, Millipore, Cat. no. AB16901, RRID: AB_11212200<br>Alex Fluor 594 donkey anti-rabbit IgG, Molecular Probes, Cat. no. A-21207, RRID: AB_141637<br>Alex Fluor 488 donkey anti-goat IgG, Molecular Probes, Cat. no. A-11055, RRID: AB_142672<br>Alex Fluor 488 donkey anti-chicken IgY, Jackson ImmunoResearch, Cat. no. 703-546-155, RRID: AB_2340376
Validation	All antibodies were either validated by the manufacturers, or by the use of knockout animals for gustducin, or by the use of blocking peptides for Plcβ2.

Eukaryotic cell lines

Policy information about cell lines

Cell line source(s)	Human embryonic kidney 293 (HEK293) PEAKrapid cells, ATCC, Cat. no. CRL-2828
Authentication	Authenticated by Manufacturer.
Mycoplasma contamination	Tested negative for mycoplasma by Manufacturer.
Commonly misidentified lines (See ICLAC register)	n/a

Animals and other organisms

Policy information about studies involving animals; ARRIVE guidelines recommended for reporting animal research

Laboratory animals	These mouse strains were used:<br>Wild-type mice (C57BL/6J background) from Jackson Lab., available at Monell Chemical Senses Center.<br>Gnat3-/- mice (C57BL/6J background) available at Monell Chemical Senses Center (Wong et al., 1996)<br>Pou2f3-/- mice (C57BL/6J background) from Dr. Ichiro Matsumoto, available at Monell Chemical Senses Center (Matsumoto et al., 2011)<br>ChAT-GFP mice (FVB/N background) from Sukumar Vijayaraghavan, available at Monell Chemical Senses Center (Grybko et al., 2011)<br>TrpM5-GFP mice (C57BL/6J background), available at Monell Chemical Senses Center (Clapp et al., 2006)
Wild animals	Wild animals not involved.
Field-collected samples	Samples collected from field not involved.
Ethics oversight	All animal experiments were performed in accordance with the National Institutes of Health guidelines for the care and use of animals in research and approved by the Institutional Animal Care and Use Committee at Monell Chemical Senses Center (ACC# 1184).

Note that full information on the approval of the study protocol must also be provided in the manuscript.
